# The Use of Raw Meat in the Treatment of Phthisis

**Published:** 1905-01-21

**Authors:** 


### THE USE OF RAW MEAT IN THE TREATMENT OF PHTHISIS.

It is generally conceded that one of the chief factors in the successful treatment of consumption is the proper regulation of the diet, and there is a consensus of opinion in favour of a diet which is rather in excess of that which the patient would need in health, consisting of food which is easily assimilated, and which includes a plentiful amount of fat, usually in the form of fresh milk. But to this Dr. R. W. Philip makes what he considers to be a most important addition to the dietetic treatment of tuberculosis. In the current number of the *Practitioner*, under the title of Zomotherapy, he calls attention to the excellent results which follow the systematic continued administration of raw meat in the treatment of consumption and other wasting diseases. For 15 years Dr. Philip has made use of raw meat in treating phthisical patients, and since the publication of Héricourt and Richet's researches four years ago, he has made the exhibition of raw meat a routine procedure. Héricourt and Richet found, in the case of dogs artificially inoculated with tuberculosis, that all such animals fed in the ordinary way speedily underwent progressive emaciation, while animals fed on raw meat in one or other form, in place of becoming thinner actually put on considerable weight. They found, moreover, that the muscle-juice expressed from the meat was as efficacious as the meat itself. Cooked meat was of very small value in comparison with raw meat. These observations have since been confirmed and amplified by others, and it has been demonstrated that the good effects of zomotherapy are not confined to animals which by nature are carnivorous, for tuberculous fowls show the same benefit. Observations on man showed that the administration of raw meat caused leucocytosis and a marked increase of hæmoglobin. Digestive leucocytosis was remarkably increased, amounting in several instances to more than double the digestive leucocytosis occurring after a meal of cooked meat. Clinical evidence has proved that the benefits of zomotherapy in consumptive patients is no less remarkable than in the lower animals. The chief objection to the treatment is the repugnance which raw meat naturally arouses, but Dr. Philip affirms that patients very quickly get over this preliminary distaste when they realise, as they speedily do, the advantages of the treatment, and as time goes by they take the raw meat with avidity. There is one other theoretical objection—namely, the possibility of introducing intestinal parasites with the raw meat. This danger is not a likely one if reasonable care be taken in the selection of meat.

### Method of Giving the Raw Meat.

The forms in which Dr. Philip has employed raw meat are as follows:—(1) Pounded raw meat, *i.e.* finely-minced or bruised beef (mutton was seldom used) slightly seasoned with salt, etc., according to taste, and served cold or gently warmed throughout—say half a pound twice or three times daily. The meat should be used perfectly fresh, and if possible obtained direct from the slaughter-house. (2) Beef-juice prepared as follows:—Extract half a pound of meat in half a pint of cold water, to which half a teaspoonful of salt has been added, for 1½ to 2 hours at 100° Fahr. Express the liquid through a cloth, and serve. Or the juice may be expressed from the meat directly without the addition of water. In either case the meat-juice must be freshly prepared before use, for it speedily undergoes changes which detract from its value and tend to cause irritation of the gastro-intestinal tract. (3) Raw-meat soup, prepared as follows:—Take half a pound of finely-minced fresh meat and mix in a bowl with sufficient milk to produce a thick uniform paste. Immediately before serving add half a pint of milk at 150° Fahr. In place of the milk, the soup may be made in similar fashion with stock of beef, chicken, or veal. (4) Dr. Philip includes raw eggs in zomotherapy. The eggs should be new laid and should not be switched or mixed with milk or other ingredient, apart from being sprinkled with salt or pepper. The patient may preface his meals with one, two, or three raw eggs.

In cold weather the chill should be taken from all these by gentle exposure to warmth immediately before use.

